# A Previsit Mobile Health App (Health-E You/Salud iTu) for Male Adolescents to Promote Sexual and Reproductive Health Care Receipt: Protocol for a Randomized Controlled Trial

**DOI:** 10.2196/77780

**Published:** 2025-10-15

**Authors:** Arik V Marcell, Annie D Smith, Sofia Osio Smith, Morayo Akande, Shelby Rohlff, Renata Arrington-Sanders, Kathleen Tebb

**Affiliations:** 1 Department of Pediatrics School of Medicine Johns Hopkins University Baltimore, MD United States; 2 Department of Population, Family and Reproductive Health Bloomberg School of Public Health Johns Hopkins University Baltimore, MD United States; 3 Department of Pediatrics Children's Hospital of Philadelphia Philadelphia, PA United States; 4 Department of Pediatrics University of California, San Francisco San Francisco, CA United States

**Keywords:** sexual health, reproductive health, primary care, mobile health, male adolescents

## Abstract

**Background:**

Male adolescents have significant sexual and reproductive health (SRH) needs and, despite the existence of national guidelines, their SRH care receipt remains poor. Technology-based interventions have been shown to successfully support adolescents’ SRH; however, they have primarily focused on female individuals. To date, no such solutions support comprehensive SRH care delivery for male adolescents in primary care settings. Health-E You/Salud iTu (Health-E You) is a mobile web app that was originally designed for female youth and has proven to be effective in improving contraceptive care delivery and use. Adolescents access the app ahead of a clinic appointment, which contains initial screening questions, interactive knowledge items, and contraception decision support with tailored recommendations based on the users’ input. The app then provides clinicians with a summary of patients’ recommended SRH care.

**Objective:**

This study aims to adapt Health-E You for use with diverse groups of male adolescents presenting for care. This protocol describes the development, testing, and evaluation of Health-E You for male youth.

**Methods:**

This study consists of 2 stages. Stage 1 involves formative research, design, and user testing of Health-E You for male adolescents. Specifically, we implemented a youth-centered design process, using multiple rounds of qualitative data collection and engaging youth and clinician advisors to design the Health-E You app from April 2023 through April 2024. From May 2025, for approximately 6 months, we are conducting beta testing of the app with youth and its output with clinicians. Transcripts of interviews and focus groups from stage 1 will be analyzed using deductive and inductive thematic analysis to identify knowledge and skill gaps for the app to address as well as desired app features. Quantitative data will be analyzed using descriptive statistics. Stage 2 will involve a stepped-wedge cluster randomized controlled trial with 2752 participants in 8 primary care settings across the United States to assess the impact of Health-E You on sexually active male adolescents’ SRH care receipt and condom use. Data will be analyzed with an intention-to-treat approach using separate mixed models for each study outcome with a random intercept to reflect clustering of patients within clinics, adjusted for clinic size.

**Results:**

Results will include qualitative data on male adolescents’ and clinicians’ perspectives on SRH care receipt and delivery, respectively, and their preferences for app design. User testing will provide qualitative and quantitative data on the feasibility, acceptability, and usability of Health-E You in clinical settings. The efficacy trial will evaluate the extent to which the app improves SRH care receipt and condom use among sexually active male adolescents.

**Conclusions:**

The findings from this study will inform future technology-based interventions for male adolescents and improve male adolescents’ SRH care receipt in primary care.

**Trial Registration:**

ClinicalTrials.gov NCT06525064; https://www.clinicaltrials.gov/study/NCT06525064.

**International Registered Report Identifier (IRRID):**

PRR1-10.2196/77780

## Introduction

### Background

Male adolescents in the United States experience high rates and key determinants of sexually transmitted infections (STIs), HIV, and unintended partner pregnancy, with male adolescents belonging to racial, ethnic, and sexual minority groups being disproportionately impacted [1-3]. National guidelines recommend primary care clinicians deliver a comprehensive sexual and reproductive health (SRH) care package to all adolescents, including male adolescents. This care package includes but is not limited to preventing, screening for, and treating STIs and HIV; preventing unintended pregnancy and reproductive system–related cancers; and counseling about healthy sexual development and relationships [4-6]. However, male adolescents’ SRH care receipt remains poor, with far fewer male adolescents being asked about and counseled on their sexual behavior and receiving family planning–related care compared to female adolescents [7-10]. One study showed that only 1 in 10 male adolescents received the recommended SRH care package and, when provided, care focused mainly on STIs and HIV prevention and treatment, with little emphasis on family planning [10].

Despite the need for interventions that facilitate SRH care delivery to male adolescents, we are not aware of any clinic-based interventions designed to support male adolescents’ comprehensive SRH care receipt. Most clinic-based SRH interventions have been designed for female adolescents to support birth control use, STI testing, and condom use negotiation [11-15]. While female-focused approaches are well justified, addressing male adolescents’ SRH needs is important for improving outcomes for *all* adolescents [16]. Existing interventions for male adolescents have focused on singular topics, such as condom use or preexposure prophylaxis for HIV, rather than the full SRH care package [17-21]. Comprehensive approaches that engage male adolescents of all genders and sexualities presenting for care are needed [22,23]. Continuing to overlook male adolescents’ SRH needs not only fails to meet their health needs but also compromises their partners’ health [6].

Technology-based interventions such as websites and mobile apps have been shown to successfully promote adolescents’ SRH [17-20]. Such interventions allow for interactive designs that can be tailored to meet adolescents’ needs while ensuring fidelity. Technology use is also ubiquitous among adolescents. They like the privacy it offers [24]; it improves their disclosure of sexual health risk behaviors [25,26], and it delivers information in ways that are perceived to be less judgmental than directly from clinicians [25-27]. While many male adolescents share that they want to talk with their clinicians about SRH, they prefer their clinician to initiate these discussions [28]. At the same time, clinicians often lack the time to have in-depth SRH conversations with male patients or feel uncomfortable doing so. The use of a technology-based intervention with male adolescents before a clinic visit may overcome some of these barriers for both adolescents and clinicians.

Health-E You/Salud iTu (Health-E You) is an interactive and individually tailored mobile web app that was originally developed to support sexually active female adolescent patients in selecting a contraceptive method that matches their personal needs and preferences and discussing contraception with their clinician. The app also prepares clinicians by sending them a confidential patient summary before the face-to-face visit. In a cluster randomized controlled trial, Health-E You significantly improved female patients’ contraceptive knowledge, self-efficacy, and use of effective nonbarrier contraceptive methods [14]. While implementing Health-E You, adolescents, clinicians, schools, and community partners voiced the need for the development of an equivalent app to use with male patients.

### Objective

This study protocol summarizes our approach to address vast research and care delivery gaps for male adolescents by adapting Health-E You for use with diverse groups of male adolescent patients presenting to primary care. This protocol describes the evaluation of the feasibility and acceptability of the Health-E You app; it also describes evaluating the app’s efficacy on male adolescent patients’ SRH knowledge, self-efficacy, and care receipt using a cluster randomized controlled stepped-wedge design as compared to usual care.

## Methods

### Study Design

**Figure 1:**
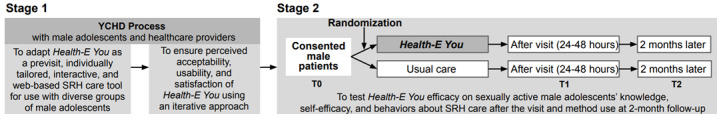
Overview of the study procedures. SRH: sexual and reproductive health; T0: baseline; T1: after clinic visit; T2: 2 months after clinic visit; YCHD: youth-centered health design.

This study consists of 2 stages (Figure 1). Stage 1 involves formative research with adolescents and clinicians to inform Health-E You app updates for use with male adolescent patients. Stage 2 involves conducting an efficacy trial to determine the impact of the updated Health-E You app on sexually active male adolescent patients’ SRH knowledge, self-efficacy, contraceptive method behaviors, and care receipt. The formative work took place from April 2023 through April 2024, and the efficacy trial began in November 2025 and will last approximately 2 years. This study used human-centered design principles to create a youth-centered final product with input from male adolescents and clinicians to ensure the app supports addressing males’ SRH. Separate from our formative research participants, described subsequently in Stage 1 Formative Research and User testing, this study is also using design team advisory groups (DTAGs) of gender-diverse male adolescents and clinicians to support the design of app content, features, and messaging; research instrument development; and recruitment strategies. This study is guided by the SPIRIT (Standard Protocol Items: Recommendations for Interventional Trials) checklist (Multimedia Appendix 1) [29].

### Ethical Considerations

This study has been approved by the Johns Hopkins Institutional Review Board (IRB00424416). In stage 1, eligible clinicians and male adolescents aged 18 years or older provided oral consent; parental oral consent and minor assent were obtained for eligible persons younger than 18 years. Male adolescent participants who provided consent and enrolled received a gift card of US $35, and clinicians received US $50 for their time after participation. For app testing, male adolescent participants who provided consent and enrolled received a gift card of US $50, and clinicians received US $50 for their time after participation. Stage 2 participants receive US $10 after completing the baseline survey and US $20 after completing each follow-up survey, totaling US $50 if they complete all 3 surveys. As part of our quality control process monitoring*,* we also have an institutional review board–approved protocol (IRB00454050) to collect online surveys before and after the intervention from clinicians and staff to provide contextual information about the trial. Each clinician or staff participant will receive a gift card of US $20 for their time after completing each survey. The protocol and consent procedures involve maintaining the privacy and confidentiality of all research subjects' data and/or identity. Survey data collected and stored through the secure Health Insurance Portability and Accountability Act (HIPAA)-compliant data management software will only be accessed by research staff. Each participant will have a unique identifier to link data between surveys and the app; participant contact information will be stored in a separate protected location that cannot be downloaded, with access limited to the research staff responsible for participant follow-up.

### Stage 1: Formative Research and User Testing

#### Formative Research Participants and Study Setting

Male adolescents and clinicians participated in virtual focus group discussions and interviews, respectively, to inform Health-E You app updates for male adolescents. Male adolescents of any gender could participate if they were of male sex; aged 15 to 24 years; English or Spanish speaking; and able to provide informed consent and recruited via local and national youth advisory boards, youth-serving organization listservs, targeted social media posts, and referrals by enrolled participants. Clinicians could participate if they had an advanced clinical degree (eg, nurse practitioner, physician); currently practiced in the United States; served at least 10% male adolescent patients; and were recruited via professional listservs, coalitions, and conferences.

#### Formative Data Collection

Focus group discussions with male adolescents explored perceptions and prioritization of different SRH topics across 3 sessions, feedback on app content using draft app pages (eg, information on condom use, STI and HIV prevention, healthy relationships, family planning), and the app’s look and feel after reviewing an updated interactive prototype developed based on findings from earlier sessions. Sessions were facilitated by a trained research member using a semistructured interview guide and conducted from April 2023 to February 2024. Each focus group session consisted of 2 to 5 participants, was conducted in English or Spanish, was stratified by age (aged 15-17 and 18-24 years) and gender identity (cisgender and gender expansive), and lasted approximately 60 to 90 minutes.

Interviews with clinicians explored perceptions of male adolescent patients’ sexual health needs, standard SRH care delivered, and areas where the Health-E You app could support SRH care delivery to male adolescents. Before the interview, clinicians completed a brief survey about their training history and confidence in and implementation of SRH care delivery to male adolescents. Interviews were conducted from May 2023 to March 2024 by a trained research member using a semistructured interview guide and lasted approximately 60 to 75 minutes.

We conducted qualitative data collection until data saturation was reached—the point at which additional data collection does not produce new information or themes [30,31]. All qualitative data were recorded and transcribed. Findings were used to develop the Health-E You app’s beta version programming by a third party.

#### App User Testing

We engaged our male youth DTAG members to test the app’s beta version, in which they worked via the app and provided feedback on its content, design, and flow. We used this feedback to inform app refinements.

We are subsequently conducting acceptability, usability, and satisfaction testing with 40 male adolescent patients and 10 clinicians. This pilot testing of the app started in May 2025 and will last approximately 6 months. Inclusion criteria for adolescents of any gender include male sex, aged 13 to 21, English or Spanish speaking, and able to provide informed consent. For this testing, eligible youth complete a brief baseline survey; then use Health-E You before their clinic appointment; and after their visit, complete an immediate post measurement survey (Table 1) and a debrief about their Health-E You experience to assess app acceptability, usability, and satisfaction, and any explanations for low app ratings [32-34]. Clinicians are asked to review and provide feedback on the app, including the app-generated patient summaries. Debrief sessions last approximately 30 to 45 minutes for male adolescents and 20 to 30 minutes for clinicians and are facilitated by a trained research member; the sessions are audio recorded and transcribed verbatim.

**Table 1:** Measures used in app user testing^a^ and efficacy trial^b^.

Variables			Items, n		Content		α coefficient		T0^a,b,c^		T1^a,b,d^		T2^b,e^
**Outcomes**													
	Knowledge about contraception methods	22		A higher score indicates higher knowledge regarding male or female contraception methods [35,36]		0.77		✓		✓		✓	
	Self-efficacy regarding contraceptive method use	7		A higher score indicates greater confidence in male method contraceptive use [37]		0.70		✓		✓		✓	
	Self-efficacy talking to a clinician	5		A higher score indicates greater self-efficacy in talking to a clinician [38]		0.83		✓		✓		✓	
	Contraceptive method use behaviors	15		Condom use at last sex (vaginal or anal); frequency and consistency of condom use during sex (vaginal or anal) in the past 2 months; female birth control method use at last sex (vaginal); reasons for condom use or nonuse at last sex [39,40]		—^f^		✓				✓	
	SRH^g^ care receipt	17		Sexual history, reproductive life plan, STI^h^ and HIV tests, pregnancy, and contraception history; condom and lubrication materials; SRH vaccine history; counseled on pregnancy prevention, contraception, EC^i^, condoms, and PrEP^j^ or PEP^k^; follow-up visit scheduled or kept [10]		—		✓		✓		✓	
	Follow-up visit	3		Intention to see clinician; visit occurred; reason visit did not occur		—				✓		✓	
**Possible moderators**													
	Sexual behavior history	12		Ever had sex (vaginal or anal); sex or gender of sex partners; number of partners in past 2 months		—		✓				✓	
	Reason for visit	1		For example, visit reasons was for well visit, sports physical, sickness, STI screen, etc [10]		—		✓		✓		✓	
**Background factors**													
	Participant background and demographics	17		Age, race and ethnicity, gender, sexual orientation, grade, self-rated mental health, parental high school completion; SRH information sources; perceived quality of SRH education from parents or school; past STI and HIV history, reproductive life plan, and partner pregnancy history; number of children [10]		—		✓				✓	
	Health care visit factors	5		About clinician—sex and time alone with clinician; about visit—perceived visit satisfaction and quality care receipt [41]		—		✓		✓		✓	
**Health-E You** **app** **process measures**													
	Acceptance	8		A higher score indicates greater app acceptance [33,34]		0.90				✓			
	Usability	12		A higher score indicates greater app usability [32]		0.98				✓			
	Satisfaction	1		A higher score indicates greater app satisfaction [33]		—				✓			
	App use data	—		Amount of time spent on app content, completion, and rating		—				✓		✓	

^a^Stage 1 user testing.

^b^Stage 2 efficacy trial.

^c^T0: baseline.

^d^T1: after clinic visit.

^e^T2: 2 months after clinic visit.

^f^Not available.

^g^SRH: sexual and reproductive health.

^h^STI: sexually transmitted infection.

^i^EC: emergency contraception.

^j^PrEP: preexposure prophylaxis.

^k^PEP: postexposure prophylaxis.

#### Formative Statistical Analysis

Collected qualitative data are being analyzed using a pragmatic approach adapted from inductive and deductive thematic analysis [42]. First, 3 study team members review transcripts to familiarize themselves with the data and then code together 2 to 3 transcripts to generate initial codes—based on discussion, interview guides, and emergent data patterns—and reach consensus on a codebook. Team members divide the remaining coding of transcripts, which is then reviewed by another team member. Coding questions or inconsistencies are resolved through weekly group discussions, with oversight from a senior team member, in which the team identifies, reviews, and names emergent themes related to app content, functionality, and design specifications.

We descriptively analyze quantitative data on perceived app acceptability, usability, and satisfaction and estimate the prevalence of agreement for each item within each scale (disagree and strongly disagree versus agree and strongly agree) using Stata 18 (StataCorp LLC) [43]. Any items that receive an agreement rating of less than 75% will be reviewed by the study team and DTAG members, alongside corresponding qualitative feedback, to identify possible areas of app improvement. We will then make these final improvements to the app before efficacy testing.

### Stage 2: Efficacy Trial

#### Efficacy Trial Setting

We will conduct the efficacy trial of Health-E You at 8 primary care settings that serve male adolescents aged 13 to 21 years from November 2025 for approximately 2 years. We recruited participating clinics through professional medical organization outreach and existing relationships. This trial has been registered on ClinicalTrials.gov (NCT06525064).

#### Stepped-Wedge Evaluation Design

We will evaluate the app’s efficacy using a cluster randomized controlled stepped-wedge design, a one-way crossover design in which all clinics will begin the study in the control phase and then transition to the Health-E You intervention [44]. This design was chosen because it allows all sites to receive the intervention over the study course. Clinics will be stratified by adolescent patient population size (large versus small) and randomized into clusters within each stratum, which will dictate when they transition to the intervention phase during the 28-month study period.

#### Participants

Patients in participating clinics will be eligible to participate in the trial if they are male, are aged 13 to 21 years, report having had vaginal or anal sex in the previous 12 months, are able to provide informed consent, and if their preferred language is English or Spanish.

#### Efficacy Trial Procedures

Consent procedures for this clinic-based study will follow state minor consent laws, which allow minors to provide consent for SRH care [45,46]. This approach upholds minor participants’ right to confidential SRH care, which is codified in each participating state’s minor consent laws and reiterated in national and federal clinical guidance for adolescent SRH care [4,5]. At each clinic, staff will share study materials (ie, flyers, banners, handout cards) with potentially eligible patients presenting for care that feature a QR code to the study’s eligibility survey, which can be completed on any device (eg, mobile phone, tablet, computer). Eligible adolescents will be electronically directed to a multimedia consent form containing an embedded video that describes the study’s purpose and design, including the number of surveys, estimated time requirements, remuneration, associated participation risks, expectations and implications of study participation, and the right to withdraw at any time without implications for their care. The video also includes a brief true or false quiz to reinforce important consent components and information on how to contact study team members (Multimedia Appendix 2).

Participants who provide consent will then be electronically autodirected to a brief baseline survey. When clinics are in the intervention phase, participants will then be autodirected to the Health-E You app after baseline survey completion. Using contact information collected at enrollment, participants will be sent a follow-up survey 48 hours after their clinic visit and then a second follow-up survey 2 months later. We use a HIPAA-compliant survey and data management software to manage all participant enrollment, data collection, and follow-up data.

#### Health-E You Intervention Condition

**Figure 2:**
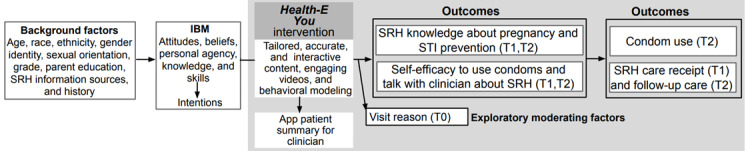
Conceptual framework of Health-E You intervention for male adolescents. IBM: Integrated Behavioral Model; SRH: sexual and reproductive health; STI: sexually transmitted infection; T0: baseline; T1: after clinic visit; T2: 2 months after clinic visit.

Health-E You leverages mobile web technology to privately ask patients standard screening questions about their SRH before their clinic visit; provide needed SRH information and skill development activities; and then subsequently share a patient summary of SRH needs, concerns, questions, and recommended SRH care with clinicians based on their responses. The design of Health-E You is informed by the integrated behavioral model, which asserts that behavior (eg, condom use and SRH care use) is influenced by core constructs of behavioral attitudes, perceived norm beliefs, and personal agency (Figure 2) [47]. Specifically, Health‐E You aims to improve male adolescents’ SRH knowledge and self-efficacy to use condoms and talk with a clinician about SRH, which collectively may result in increased SRH care receipt during their clinic visit and subsequent condom use behaviors.

Similar to the app’s original version for female adolescents, male adolescents using the Health-E You app are guided through an initial question set related to sexual behavior, sexual health history, and family planning goals (Multimedia Appendix 3). Users are subsequently directed to a core condom use and other STI and HIV prevention methods (eg, vaccines and preexposure prophylaxis) content pathway—designed to be completed in 7 to 10 minutes. The core content is multimedia and interactive to accommodate different engagement preferences and reinforce the app’s messages (eg, brief condom demonstration video, interactive activity on condom use steps, animated informational videos provided by Amaze [48], videos from real clinicians and young people developed for this study, written content). Based on users’ family planning goals and interests, users may also receive content on hormonal pregnancy prevention methods and, for participants expecting a child, ways to support a pregnant partner or newborn.

After completing core content, each user is presented with a summary of recommended topics to discuss with their clinician and invited to select additional SRH topics and submit questions they would like to discuss with their clinician during their visit. Users are given the option to send this patient summary to their clinician securely by email or local printing at the clinic, based on the clinic’s preferred workflows. All Health-E You app data are programmed and hosted on the Drupal web-based interface and securely stored in the HIPAA-compliant Salesforce Platform (Salesforce Inc), which adheres to strict data security standards, including regular security updates.

#### Control Condition

When clinics are in the control condition, participants will receive care as usual.

#### Study Measures and Outcomes

In the baseline survey, participants are assessed for past SRH care receipt [10]; sexual behavior, including condom use [39,40]; SRH knowledge [35,36]; self-efficacy related to condom use [37] and talking to clinicians about SRH [38]; and background characteristics [10] (Table 1). At the first follow-up survey (48 hours after the visit), participants were assessed for SRH care receipt at the clinic visit, visit satisfaction, quality care receipt [41], and SRH knowledge and self-efficacy and, for participants in the intervention phase, acceptability [33,34], usability [32], and satisfaction [33] using the Health-E You app. At the second follow-up survey 2 months later, participants are assessed for the same baseline measures, including method use behaviors, and any additional app engagement (Table 1).

#### Retention

Strategies for participant retention from baseline through the follow-up surveys include participant remuneration, collecting multiple contacts at baseline and subsequent follow-ups (ie, phone number, email, etc), check-ins with participants between surveys, advising and providing US $5 gift cards to participants contacting us with changes in contact information, and sending reminder messages before conducting follow-up procedures [14,40].

#### Power and Sample Size

We generated power analyses using NCSS PASS 16 to compute minimum detectable effect sizes for condom use at last intercourse [40]. Assuming 2064 participants at the 2-month follow-up (86 participants per clinic per assessment), α of .05, power of .80, and an intracluster correlation coefficient of 0.06 (calculated from previous Health-E You data with female individuals [14]) with a baseline prevalence of 61.1% condom use at last intercourse [40], the minimum detectable difference is 12.2 percentage points, which translates to an effect size (h) of 0.26, just above the accepted threshold for a small effect size of 0.20. A baseline sample of 2752 participants (approximately 115 participants per clinic per step) will be recruited to account for 79.9% (2201/2752) retention at immediate follow-up (48 hours) and 75% (2064/2752) retention at 2-month follow-up.

#### Efficacy Trial Statistical Analysis

The main effects of the Health-E You intervention are being estimated using an intention-to-treat approach using separate mixed models for each study outcome, with a random intercept to reflect clustering of patients within clinics and adjustment for clinic size [49]. Each model estimates differences between the intervention and control arm in the change in each study outcome between baseline and each follow-up. This type of analysis allows for examining (1) within-individual change over time in an outcome, accounting for dependency among observations within individuals by modeling within-person error [50]; (2) between-individual variation over time [51]; and (3) between-subjects effects based on group assignment to identify significant differences in Health-E You versus usual care. We will also assess the potential moderating effect of patient demographics and adolescents’ visit reason, which may influence SRH care receipt, on the study’s outcomes. We are addressing incomplete data with maximum likelihood and multiple imputations [52-54]. The use of these missing replacement strategies does not mitigate loss of statistical power but does minimize bias because of attrition [55].

#### Quality Control Process Monitoring

The investigative team at the Johns Hopkins School of Medicine, which is serving as the coordinating site for this multi-site randomized controlled trial, is serving as the data management team. This team will communicate any important protocol modifications to relevant parties, as appropriate, and in conjunction with the institutional review board. The investigative team comprises experts in adolescent health, male health, and evaluation research. We are using the World Health Organization’s Monitoring and Evaluating Digital Health Intervention guidelines to inform this study’s process monitoring of system factors, intervention quality, and intervention engagement [56]. We will assess technical functionality and stability through periodic user tests by the study team and routine app data monitoring to confirm the app is functioning as expected. We will assess intervention quality through the 48-hour follow-up survey, which will include questions on usability, acceptability, and satisfaction, including an overall app rating. We will track intervention engagement by monitoring whether participants complete core Health-E You content, opt in rates to share the patient summary with their clinician, time spent on Health-E You, and viewed content. We will provide monthly reports to participating clinics on these metrics and participant enrollment, which will provide an opportunity to gather clinic feedback to understand and address barriers with participant recruitment or sharing the patient summaries with clinicians. We will also collect online surveys before and after the intervention from clinicians and staff at each clinic about clinic workflows and visit times and postintervention about clinicians’ perceived acceptability and usability of and satisfaction with Health-E You to provide contextual information about the trial.

#### Safety Monitoring

This study is guided by an internal data safety monitoring plan that includes staff training and monitoring related to participant privacy, confidentiality, and identification; response; and reporting of different possible events, adapted from recommendations by Horigian et al [57] (Multimedia Appendix 4). The data safety monitoring plan is managed by the principal investigator with oversight from a designated monitor external to the study team. This study is protected under a certificate of confidentiality from the National Institutes of Health that protects the research and Health-E You app data from subpoena.

## Results

Findings from our stage 1 formative research and user testing will provide insights into the content, design, feature needs, and preferences of diverse male adolescents and clinicians regarding the development of an app to support young male adolescents’ SRH care receipt and behaviors.

Findings from the stage 2 efficacy trial of Health-E You will provide important information on the effects of the Health-E You app on male adolescent patients’ SRH knowledge, self-efficacy, and care receipt immediately after their clinic visit; condom use behaviors and additional SRH care receipt 2 months later; and acceptability, usability, satisfaction, and engagement with the app among intervention participants. Our analysis will also provide an indication of whether Health-E You produced different effects among participating male adolescents from varying backgrounds and presenting to care for different reasons. Results will be shared with participating clinics, updated on ClinicalTrials.gov (NCT06525064), and submitted for review to peer-reviewed journals.

## Discussion

### Anticipated Findings

There is a significant need for innovative approaches that support SRH care delivery for male adolescents. Our adaptation of the Health-E You/Salud iTu mobile web app for male adolescents offers a scalable, high-fidelity tool that primes both male adolescents and clinicians to discuss SRH, which adolescents can return to on their own time for further education and skill building. If Health-E You is found to be efficacious for male adolescents, it will provide a unique opportunity to engage male adolescent patients in much-needed SRH care.

Our iterative, human-centered design approach to adapt Health-E You for male adolescents is a strength of this study because it ensures that the app’s content and features are aligned with the needs and preferences of both male adolescents and the clinicians who deliver their care. Human-centered design principles that we are implementing, such as continuous engagement, rapid prototyping, and iterative feedback, have been increasingly recognized as valuable for the design of SRH and other health interventions [58]. Furthermore, recent literature reviews suggest that engaging intended beneficiaries produces more relevant interventions and higher quality research that is more broadly disseminated [59-62].

The cluster randomized controlled stepped-wedge design in the efficacy trial of Health-E You will enable all participating clinics to receive the intervention while still maintaining rigor in estimating its effects on adolescents’ SRH. If shown to be efficacious for male adolescent patients, Health-E You will likely be of use for male patients in similar settings across the United States and other clinical settings where currently no comparable strategy exists (eg, school-based health clinics, community clinics, college health services) and could be adapted for use in nonclinical settings (eg, school health classes, foster care, juvenile justice, and community-based organizations). The use of web-based technology is easy to scale up and sustain with regard to cost [63]. Ultimately, this study will contribute to advancing quality SRH care receipt for diverse groups of male adolescents and fill a critical gap that has existed for far too long without any attention.

### Limitations

This study is not without limitations. Findings from our stage 1 formative research and user testing are not generalizable to all US male adolescents. This is a limitation inherent to most qualitative research studies, which prioritizes the richness and depth of data collected from participants with lived experience relevant to the research question rather than representative sampling [64]. In our stage 2 efficacy trial, we will recruit participants in primary care clinics across multiple states with diverse patient populations in an effort to approximate a sample that is more representative of male adolescents seeking primary care. All study participants will be volunteers and may exhibit selection biases that will be assessed and, if identified, accounted for analytically. Retention may also be an issue in the efficacy trial, though recommended strategies, such as the use of online surveys, incentives, and regular communication, will be implemented [65]. Contamination is theoretically possible; however, it seems unlikely given our clinic-level randomization and stepped-wedge design in which all clinics will start with the control condition and then transition to intervention receipt. There are also limitations to self-reported measures. It is possible responses may differ from actual behavior, although the use of such an approach is common in research such as this and well documented to be valid when assessed privately [66]. Despite these limitations, study findings will provide meaningful information on the potential for Health-E You to address key behavioral determinants of SRH and care for male adolescents.

### Conclusions

This study protocol is intended to evaluate Health-E You by using a rigorous randomized stepped-wedge design to determine its feasibility, acceptability, and efficacy. If found to be efficacious, Health-E You will assist in improving SRH outcomes and care receipt for male adolescents as part of primary care.

## Appendix

Multimedia Appendix 1 SPIRIT checklist.

Multimedia Appendix 2 Link to Health-E You informed consent video for efficacy trial.

Multimedia Appendix 3 About the Health-E You app.

Multimedia Appendix 4 Monitoring of adverse events in Health-E You efficacy trial (Stage 2).

Multimedia Appendix 5 Peer-review report from Health Services: Quality and Effectiveness Study Section (HSQE) (National Institutes of Health, USA).

## Abbreviations

DTAG: design team advisory group
HIPAA: Health Insurance Portability and Accountability Act
SPIRIT: Standard Protocol Items: Recommendations for Interventional Trials
SRH: sexual and reproductive health
STI: sexually transmitted infection

## Data Availability

The datasets generated or analyzed during this study will not be publicly available to protect sensitive information but will be available through restricted or controlled access from the corresponding author on reasonable request.
