# Supplementary material for: A Previsit Mobile Health App (Health-E You/Salud iTu) for Male Adolescents to Promote Sexual and Reproductive Health Care Receipt: Protocol for a Randomized Controlled Trial
Source: JMIR Res Protoc. 2025 Oct 15;14:e77780. doi: 10.2196/77780 (PMC12572748; doi:10.2196/77780)
Supplement: Multimedia Appendix 2 [file resprot_v14i1e77780_app2.docx]

**Supplement: Multi-media**

**Link to Health-E You Informed Consent Video for Efficacy Trial:**

<https://vimeo.com/1115270811/bc7a17df7c?share=copy>
