# Supplementary material for: A Previsit Mobile Health App (Health-E You/Salud iTu) for Male Adolescents to Promote Sexual and Reproductive Health Care Receipt: Protocol for a Randomized Controlled Trial
Source: JMIR Res Protoc. 2025 Oct 15;14:e77780. doi: 10.2196/77780 (PMC12572748; doi:10.2196/77780)
Supplement: Multimedia Appendix 4 [file resprot_v14i1e77780_app4.docx]

**Multimedia Appendix 4.** Monitoring of adverse events in Health-E You efficacy trial (Stage 2).

| Event | Severity | Operational definition | Resolution | Related | Expected | Staff response or follow-up protocol |
| --- | --- | --- | --- | --- | --- | --- |
| Verbal abuse or harassment | Nonserious | Young person reports verbal abuse, harassment, or bullying from a parent, peer, partner, or others. | The event is resolved when the study staff connects with the participant and parent. | Yes or no | Yes | - Study staff review refusal text response as “conflict with parent.” - Staff will share resources with the participant and parents and recommend follow-up with the study team. |
| Psychological distress | Serious adverse event or nonserious | Participant experiences emotional discomfort in response to a stressor that is severe enough to result in harm to the participant. | The event is resolved when the clinical supervisor or other qualified professional determines that there is no further risk. | Yes or no | No | - As soon as the study staff is alerted to psychological distress in a participant, PI^a^ will be contacted. - The PI will contact the participant and may refer the participant to ED^b^ or a social worker, as appropriate. |
| Physical violence | Serious adverse event or nonserious | Participant is subjected or exposed to rough or injurious physical force or abuse, action, or treatment but does not require medical attention. The participant can be a witness to a violent event that involves death, serious injury, or a real threat to physical integrity. | The event is resolved when the violent event or exposure to a violent event ends. | No | No | - As soon as the study staff is alerted to any general violence, the PI will be contacted. - PI will contact the participant and may refer the participant to ED or a social worker, as appropriate. |
| Intimate partner violence (survivor or exposure) | Serious adverse event or nonserious | Participant is subjected or exposed to partner violence or abuse, including physical, verbal, or emotional abuse, regardless of requiring medical attention. | The event is resolved when the violent event or exposure to a violent event ends. | Yes or no | No | - As soon as the study staff is alerted to psychological distress in a participant, the PI will be contacted. - Active referral to the ED and to a social worker will be provided. |
| Physical or sexual abuse | Serious adverse event | Participant is a survivor of physical assault, resulting in bruises, marks, or injury requiring medical attention. This can include, but is not limited to, injury inflicted by hitting, kicking, burning, shaking, or throwing. Participant is a survivor of unwanted sexual contact or behavior. This can include, but is not limited to, attempted rape or actual rape, fondling or unwanted sexual touching, or forcing the survivor to perform sexual acts. | This event refers to a physical or sexual-abuse incident. Repeated incidents should be reported as separate events. The event is resolved when the physical or sexual abuse ends. | No | No | - As soon as study staff is alerted to any physical or sexual abuse, PI will be contacted. - PI will contact participant and may refer participant to ED or social worker, as appropriate. |
| Unstably housed | Serious adverse event or nonserious | Participant has been kicked out of or run away from home and reports that they currently do not or anticipate that they will not have a regular place to sleep. | The event is resolved when safe housing is identified. | No | No | - As soon as the study staff is alerted to any general violence, the PI will be contacted. - PI will contact the participant and may refer the participant to a social worker or youth-serving organization, as appropriate. |
| Hospitalization | Serious adverse event | Participant is hospitalized for any reason. | Resolution of the event is discharge from the hospital. This is a clear-cut incident that starts and ends with the occurrence of the event. | No | No | - As soon as the study staff is alerted to participant hospitalization, the PI will be contacted. - Enrollment status will depend on the participant’s unique circumstances. |
| Death | Serious adverse event | The participant has died. | This is a clear-cut incident that starts and ends with the occurrence of the event. | No | No | - As soon as the study staff is alerted to the death of a participant, the PI will be contacted. - Participant would be disenrolled. |
| Breach of confidentiality | Nonserious | The participant’s personally identifiable information is disclosed without authorization. | This is a clear-cut event that is resolved on the date when all parties are notified about the breach. | No | No | - If there has been a breach of confidentiality, the following people or entities should be informed: participant, IRB^c^, HIPPA^d^ office, and NIH^e^, as appropriate. |
| Arrest and release | Serious adverse event or nonserious | The participant is arrested and released from police custody, which could have resulted in the disruption of study participation. | An arrest is a clear-cut event that starts with the arrest date and is resolved with the date of release from custody. | No | No | - If study staff learns of an arrest, the length of imprisonment will be determined. If the arrest is for less than 4 weeks, the participant will remain enrolled. If it is for longer than 4 weeks, the participant will be disenrolled. |
| Imprisonment or incarceration | Serious adverse event or nonserious | The participant is imprisoned, which will result in loss of cell phone access and thus be a reason for disenrollment, as it may affect her ability to complete follow-up activities. | This is a clear-cut event that starts and ends with the imprisonment date. Therefore, the date of onset and date of resolution are the same. | No | No | - Participant would be disenrolled. |
| Other unexpected adverse event | Serious adverse event or nonserious | Any other adverse event, serious or not serious | Resolution is determined by the PI or clinical supervisor. | Yes or no | No | - If study staff is alerted to an unexpected adverse event that may affect participation in the study, the PI will be contacted. |

^a^PI: principal investigator.

^b^ED: emergency department.

^c^IRB: institutional review board.

^d^HIPPA: Health Insurance Portability and Accountability Act.

^e^NIH: National Institutes of Health.
