# Supplementary material for: A Previsit Mobile Health App (Health-E You/Salud iTu) for Male Adolescents to Promote Sexual and Reproductive Health Care Receipt: Protocol for a Randomized Controlled Trial
Source: JMIR Res Protoc. 2025 Oct 15;14:e77780. doi: 10.2196/77780 (PMC12572748; doi:10.2196/77780)
Supplement: Multimedia Appendix 5 [file resprot_v14i1e77780_app5.pdf]

1R01HD109141-01 Marcell, Arik

NEW INVESTIGATOR

RESUME AND SUMMARY OF DISCUSSION: This application from a New Investigator proposed a study to adapt the Health-E You/Salud iTu contraceptive knowledge and contraceptive care receipt web-based mobile app for use with diverse male adolescents. The study would also examine its acceptability, usability, and satisfaction among male adolescents and providers as well as its efficacy on improving male adolescents' knowledge, self-efficacy, beliefs, and behaviors about sexual and reproductive health (SRH) care. Reviewers agreed that that the proposed study is highly significant because it addresses a clear need for strategies to increase use of SRH care services by adolescent males. Rigor of prior research was strong, based on a compelling literature review. The investigative team was excellent, having complementary expertise and including the developer of the original intervention. The environment was similarly excellent and has all the necessary facilities, equipment, and resources to support the proposed scope of work. The well-described approach was also a strength. However, reviewers also identified some weaknesses. These included interdependence of study aims and potential for social desirability bias because of reliance on self-reported data. Reviewers disagreed about whether the convenience sample raised concerns about representativeness, diversity, and selection bias. The panel concluded that the strengths far outweighed the weaknesses, however, and that the proposed study would have a high impact on the field of adolescent health care.

DESCRIPTION (provided by applicant): While preventive care guidance recommends primary care providers deliver sexual and reproductive health (SRH) care to male adolescents (ages 12 and older), males' SRH care receipt is poor. Clinic-based interventions can be valuable SRH promotion tools for adolescents. However, they have mainly focused on pregnancy prevention for females, single topics (STIs, HIV) rather than the recommended SRH care package, or specific male groups (e.g., men who have sex with men) rather than inclusive of the diversity of males encompassing the full range of gender identities, sexual orientation, race/ethnicity, and risk behaviors. Computer-based approaches make it easier to consistently provide evidence-based SRH, in multiple languages, tailored to the diverse needs of all adolescents. Using such approaches before the clinic visit can also help overcome providers' barriers, such as time constraints, to improve SRH care. Yet, we are not aware of any computer-based strategy to promote recommended SRH care for diverse groups of males. Neglecting males in evidence-based SRH care fails to meet their own needs, and compromises their partners' health. Health-E You/Salud iTu is a pre-visit, individually tailored, interactive, web-based mobile intervention shown to improve contraceptive knowledge and use among adolescent females 6 months later and prime and extend providers' ability to deliver individually tailored contraceptive care to female patients. We will adapt the current Health-E You for male adolescents to assess their SRH needs; provide interactive, individually tailored, evidence-based SRH information; support SRH decision-making and visit priorities; and support providers' ability to individually tailor recommended SRH care. We will then evaluate its acceptability, usability, satisfaction, and efficacy on SRH care receipt and method use among diverse groups of male adolescents presenting for care at school-based health centers (SBHCs), by leveraging the infrastructure of a current PCORI-funded trial with 28 SBHCs in 11 states focused on assigned females sex at birth. It will also support providers in delivering the recommended SRH care package for males. In this R01 proposal, we propose to (1) adapt Health-E You as a pre-visit, individually tailored, interactive, SRH care tool for use with diverse groups of male adolescents employing a Youth-Centered Health Design Process with input from an advisory board of male adolescents and providers; (2) ensure its acceptability, usability, and satisfaction among male adolescent patients and providers in SBHCs using an iterative design approach; and (3) test its efficacy

to improve sexually active male adolescent patients' knowledge, self-efficacy, beliefs, and behaviors related to SRH care after the visit and method use 2 months later. The current proposal would be the first to examine the acceptability, usability, satisfaction, and efficacy of a pre-visit, computer-based intervention to engage sexually active male adolescent patients in SRH care and method use, where currently no such strategy exists.

PUBLIC HEALTH RELEVANCE: Male adolescents have substantial sexual and reproductive health (SRH) needs, including high rates of sexually transmitted infections, human immunodeficiency virus, cancer prevention, and unintended partner pregnancies; yet despite clinical guidelines that recommend primary care providers deliver SRH care to all male adolescents, very few males receive any singular SRH service. Computer- and clinic-based strategies have potential to address this gap, but to date, there are not any approaches that promote the recommended SRH care package for diverse groups of males presenting to care. This project will adapt Health-E You/Salud iTu, a pre-visit, individually tailored, web-based mobile app that improved female adolescents' contraceptive knowledge and contraceptive care receipt, for use with diverse male adolescents; and will examine its acceptability, usability, and satisfaction among male adolescents and providers; and its efficacy on improving male adolescents' knowledge, self-efficacy, beliefs, and behaviors about SRH care after the visit and method use 2 months later.

CRITIQUE 1

Significance: 2
Investigator(s): 2
Innovation: 4
Approach: 1
Environment: 1

Overall Impact: Consistent, widespread, and appropriate delivery of sexual and reproductive (SRH) care has broad public health implications. While delivery of SRH care is recommended for all adolescents, male adolescents are far less likely to receive it despite being at risk for acquiring sexually transmitted infections and for unintended partner pregnancy. Use of a pre-visit, individually tailored, web-based app in School Based Health Clinics has been shown to be efficacious for improving SRH care delivery, improving SRH knowledge in adolescent females, and modifying SRH behaviors. The investigators propose to adapt the Health-E You/ Salud-iTu app for use in male adolescents, assess its usability, and test its impact on SRH knowledge and behavior in a step-wedge randomized clinical trial using existing infrastructure from an ongoing PCORI-funded trial that one of the co-investigators leads. Though use of the app/ approach is not novel for SRH education given its demonstrated efficacy in female adolescents, the authors provide strong reasoning as to the need for refinements and testing in male adolescents and the novelty for its use in males. The investigative team is strong, collaborative, and has the expertise and research environment necessary to successfully complete the aims. This study has great potential to advance the knowledge base and address gaps in SRH care quality and equity, for males across racial and ethnic groups.

1. Significance:

Strengths

- Sexual and Reproduction Health (SRH) care receipt is poor for male adolescents despite recommendations for routine delivery of care.

- Male adolescents have high rates of STIs and unintended partner pregnancy; yet male adolescents are far less likely to receive SRH care compared to females.
- Improvements in SRH care has important public health implications for STI/ HIV prevention including across racial and ethnic groups.
- The development and testing of approaches to addressing SRH needs in males can improve outcomes in all adolescents.

Weaknesses

- None noted by reviewer.

2. Investigator(s)

Strengths

- The PI who qualifies as a New Investigator for this proposal has expertise in pediatric and adolescent medicine, sexual and reproductive health, health services research, behavioral sciences, and mixed-methods approaches to research. The PI has been continuously funded since 2003 with awards to explore health needs and barriers in the adolescent male population and to test strategies of behavior change.
- The PI has collaborated with other co-investigators on other projects and publications.
- Co-I Sanders brings additional expertise in the health of sexual and gender minority youth as well as expertise in mixed methodology and multi-levels strategies to address barriers to care.
- Co-I Tebb brings extensive experience in clinical trials leadership, implementation/execution for sexual and reproductive health projects and technology-based projects. Co-I Tebb developed and tested the Health-E You/ Salud-iTu app which will be used in for the trial.
- Co-I Pollack brings statistical and methodological expertise and has served as statistician and multiple trials including app-based interventions.

Weaknesses

- None noted by reviewer.

3. Innovation:

Strengths

- There are currently no known clinic-based or technology-based interventions aimed at improving delivery of SRH care to male adolescents. In this proposal, the Health-E You/ Salud-iTu app will be adapted for male adolescents and young adults and will be tested in this group.

Weaknesses

- The Health-E You/ Salud-iTu app has already been studied and proven to be effective in Latina adolescents and young adults and has already been adapted for use in adolescent females.

4. Approach:

Strengths

- The investigators will use existing infrastructure to of a PCORI-funded trial to execute the study.

- The investigators will engage key stakeholders including adolescent males to adapt the app for use in this group.
- Preliminary studies support the planned proposal including successful studies to engage groups of adolescents in SRH care, and studies demonstrating care gaps despite the interest of male adolescents in receiving care.
- Authors describe a strong foundational conceptual model informed by the Integrated Behavioral Model.
- Recruitment will reflect the diversity of SBHCs in the study.
- The investigators will used strong previously examined approaches (Youth Centered Design Process) to adapt the application for uses in the male population.
- Very clearly described selection of step-wedge design methodology to allow males at all sites to receive intervention, while using statistical methods to address potential impact of secular trends.

Weaknesses

- Completion of Aim 3 relies on successful completion of Aims 1 and 2; however, given the team's previous record in adapting the application, likelihood of successful completion of Aims 1 and 2 is very high.

5. Environment:

Strengths

- Strong research infrastructure at the Johns Hopkins Schools of Medicine and Public Health with multiple Centers to support clinical trials activities.
- At UCSF, there is sufficient space, human resources, audiovisual materials, and distribution capabilities for dissemination of information.
- Ability to use the established infrastructure developed with school-based health clinics for the PCORI funded trial is major strength.

Weaknesses

- None noted by reviewer.

Study Timeline

Strengths

- In general, very clear and well-organized reasoning for confidence in being able to adhere to the timeline as described.

Weaknesses

- One quarter (3 months) between completion of Aim 2 and enrollment in the clinical trial may be challenging if refinements must be made.

Protections for Human Subjects

Acceptable Risks and/or Adequate Protections

- Investigators describe appropriate protections of data, privacy, and protection of minors.

Data and Safety Monitoring Plan (Applicable for Clinical Trials Only):
Acceptable

- Appropriate monitoring plan given low risks associated with the intervention.

Inclusion Plans

- Sex/Gender: Distribution justified scientifically
- Race/Ethnicity: Distribution justified scientifically
- For NIH-Defined Phase III trials, Plans for valid design and analysis: Not applicable
- Inclusion/Exclusion Based on Age: Distribution justified scientifically
- Appropriate plans and reasoning for inclusion of male adolescents but with diverse enrollment by race and ethnicity. The planned enrollment table includes females. It is not clear if this is an error.

Vertebrate Animals

Not Applicable (No Vertebrate Animals)

Biohazards

Acceptable

Resource Sharing Plans

Acceptable

Budget and Period of Support

Recommend as Requested

CRITIQUE 2

Significance: 3
Investigator(s): 2
Innovation: 2
Approach: 4
Environment: 2

Overall Impact: This R01 application from a New Investigator seeks to leverage the infrastructure of a current PCORI-funded trial with 28 school-based health clinics to adapt an interactive, web-based sexual and reproductive health intervention for adolescent females to adolescent males. The investigators are well-poised to conduct this study leveraging their expertise in adolescent sexual health, mixed methods research, intervention development and evaluation, and multicenter trials using stepped wedge design. Further the Co-I developed the Health E You App initially designed for adolescent females for pregnancy prevention which will be adapted to develop comprehensive SRH to males. This study is highly innovative in its use SBHCs, which are an under-tapped area for health care

delivery, and its focus on males for SRH education. The approach is well developed and clearly described. Although Aim 3 is dependent on the successful execution of Aims 1 and 2, given the preliminary data, this seems feasible. It would have been helpful for the investigators to describe how clinicians would be receiving the summary message from the app and the study may be more robust if objective data from the EHR were also collected (e.g., STI test results, etc) so data are not just reliant on self-report.

1. Significance:

Strengths

- Although male adolescents have substantial sexual and reproductive health needs and guidelines recommend primary care physicians deliver comprehensive SRH care to all male adolescents, few males receive SRH services, and when they do, they are limited to STI/HIV care. Computer-based strategies can address this gap, but comprehensive sexual and reproductive health interventions have not previously been developed for diverse groups of males.

Weaknesses

- None noted by reviewer.

2. Investigator(s):

Strengths

- The PI is a national expert in male adolescent sexual and reproductive health care delivery and has a track record of funding and publications in this area.
- The PI is supported by Co-Is with expertise in mixed methods research, biostatistics, stepped wedge design for multicenter trials, intervention development and evaluation.
- The PI who developed Health-E You/Salud iTu app that will be adapted for males in this proposal is a Co-I.

Weaknesses

- None noted by reviewer.

3. Innovation:

Strengths

- Intervention delivery in school-based health centers is highly innovative. This is an under-tapped area for health care delivery, especially preventative health services.
- Focus on adolescent males for SRH education and service delivery – this group has been neglected in most SRH work.

Weaknesses

- None noted by reviewer.

4. Approach:

Strengths

- The conceptual model is well presented.
- The analytic plan for each aim is well described.
- The study is appropriately powered.

Weaknesses

- Aims are dependent upon each other, but since Aims 1 and 2 are developmental and iterative this is less concerning, especially because some work has already been conducted through a pilot award and the tool will be adapted rather than developed from scratch.
- Although the investigators state Aims 1 and 2 will also include Spanish-speaking participants based on inclusion criteria, they do not explicitly state whether separate focus groups will be conducted in Spanish.
- It would have been helpful for the investigators to provide where the 28 SBHCs are located to speak to generalizability.
- The investigators do not describe how the clinicians will receive the summary message from the Health-E You app once a study participant completes the intervention.
- The study would have been more robust if data could also be collected from the EHR with respect to STI test results, clinician notes, etc. so data are not reliant just on self-reported data that may be subject to social desirability bias.

5. Environment:

Strengths

- Hopkins provides an excellent environment for this proposed work through the School of Medicine as well as the School of Public Health.

Weaknesses

- None noted by reviewer.

Study Timeline

Strengths

- Well outlined.

Weaknesses

- None noted by reviewer.

Protections for Human Subjects

Acceptable Risks and/or Adequate Protections

Data and Safety Monitoring Plan (Applicable for Clinical Trials Only):

Unacceptable

- Not provided.

Inclusion Plans

- Sex/Gender: Distribution not justified scientifically

- Race/Ethnicity: Distribution justified scientifically
- For NIH-Defined Phase III trials, Plans for valid design and analysis: Not applicable
- Inclusion/Exclusion Based on Age: Distribution justified scientifically

Vertebrate Animals
Not Applicable (No Vertebrate Animals)

Biohazards
Not Applicable (No Biohazards)

Resource Sharing Plans
Acceptable

Budget and Period of Support
Recommend as Requested

CRITIQUE 3

Significance: 2
Investigator(s): 2
Innovation: 2
Approach: 4
Environment: 2

Overall Impact: Male adolescents experience high rates of STIs and HIV, partner unintended pregnancy, and related morbidity. Despite preventive care guidance recommending all male adolescents receive substantial sexual and reproductive health (SRH) care, but very few males receive any singular SRH service. This project will adapt Health-E You/Salud iTu, a pre-visit, individually tailored, web-based mobile app for improving female adolescents' contraceptive knowledge and contraceptive care receipt, for use with diverse male adolescents; and will examine its acceptability, usability, and satisfaction among male adolescents and providers; and its efficacy on improving male adolescents' knowledge, self-efficacy, beliefs, and behaviors about SRH care after the visit and method use 2 months later. With the successful of the proposed work, it will provide a web based mobile app for improving males' SRH care. Therefore, the impact is high.

1. Significance:

Strengths

- Male adolescents experience high rates of STIs and HIV, partner unintended pregnancy, and related morbidity.
- An individually tailored, web-based mobile app which can provide SRH care to male adolescents will reduce the rates of STIs and HIV, partner unintended pregnancy, and related morbidity.

Weaknesses

- None noted by reviewer.

2. Investigator(s):

Strengths

- The interdisciplinary team of adolescent health researchers with expertise in health disparities; SRH promotion, intervention, and care; implementation science; applying technology for health interventions; research with Black and Latinx adolescents; LGBTQ health; qualitative research; biostatistics; and collaborative community school-based health partnerships is a strength for the proposed study.

Weaknesses

- None noted by reviewer.

3. Innovation:

Strengths

- It is the first study to examine the acceptability, usability, satisfaction, and efficacy of a pre-visit, individually tailored, interactive, web-based mobile intervention (Health-E You/Salud iTu) to promote male adolescent patient SRH and care.

Weaknesses

- None noted by reviewer.

4. Approach:

Strengths

- Leverage current PCORI infrastructure places the research team in a unique position to address the full SRH care package for diverse groups of male adolescents.
- The proposed study will be conducted in 20 of the 28 high school SBHCs in the PCORI-funded study across 11 states and Washington, DC.

Weaknesses

- There is concern on 1) the representative 2) diversity and 3) selection bias. For example, in the Aim 2, the convenience sample was used and the composition of the 10-12 focus group with male adolescents is not provided. In the planned recruitment, it includes majority of female, and does not match the 1,866 male adolescents in Aim 3. There is a lack of information of the targeted cohort in Aim 3 regarding to the ethnicity/race distribution.
- There is insufficient data analysis plan for selection bias.

5. Environment:

Strengths

- The environment is adequate for the proposed research.

Weaknesses

- None noted by reviewer.

Study Timeline

Strengths

- The study timeline is reasonable.

Weaknesses

- None noted by reviewer.

Protections for Human Subjects

Acceptable Risks and/or Adequate Protections

- Appropriate protections of data, privacy, and protection of minors.

Data and Safety Monitoring Plan (Applicable for Clinical Trials Only):
Acceptable

- Appropriate monitoring plan.

Inclusion Plans

- Sex/Gender: Distribution justified scientifically
- Race/Ethnicity: Distribution justified scientifically
- For NIH-Defined Phase III trials, Plans for valid design and analysis: Not applicable
- Inclusion/Exclusion Based on Age: Distribution justified scientifically

Vertebrate Animals

Not Applicable (No Vertebrate Animals)

Biohazards

Not Applicable (No Biohazards)

Resource Sharing Plans

Acceptable

Budget and Period of Support

Recommend as Requested

THE FOLLOWING SECTIONS WERE PREPARED BY THE SCIENTIFIC REVIEW OFFICER TO SUMMARIZE THE OUTCOME OF DISCUSSIONS OF THE REVIEW COMMITTEE, OR REVIEWERS' WRITTEN CRITIQUES, ON THE FOLLOWING ISSUES:

PROTECTION OF HUMAN SUBJECTS: ACCEPTABLE

INCLUSION OF WOMEN PLAN: ACCEPTABLE

INCLUSION OF MINORITIES PLAN: ACCEPTABLE

INCLUSION ACROSS THE LIFESPAN: ACCEPTABLE

COMMITTEE BUDGET RECOMMENDATIONS: The budget was recommended as requested.

Footnotes for 1 R01 HD109141-01; PI Name: Marcell, Arik V

NIH has modified its policy regarding the receipt of resubmissions (amended applications).See Guide Notice NOT-OD-18-197 at https://grants.nih.gov/grants/guide/notice-files/NOT-OD-18-197.html. The impact/priority score is calculated after discussion of an application by averaging the overall scores (1-9) given by all voting reviewers on the committee and multiplying by 10. The criterion scores are submitted prior to the meeting by the individual reviewers assigned to an application, and are not discussed specifically at the review meeting or calculated into the overall impact score. Some applications also receive a percentile ranking. For details on the review process, see http://grants.nih.gov/grants/peer_review_process.htm#scoring.

MEETING ROSTER

Health Services: Quality and Effectiveness Study Section
Healthcare Delivery and Methodologies Integrated Review Group
CENTER FOR SCIENTIFIC REVIEW
HSQE
02/24/2022 - 02/25/2022

Notice of NIH Policy to All Applicants: Meeting rosters are provided for information purposes only. Applicant investigators and institutional officials must not communicate directly with study section members about an application before or after the review. Failure to observe this policy will create a serious breach of integrity in the peer review process, and may lead to actions outlined in NOT-OD-14-073 at https://grants.nih.gov/grants/guide/notice-files/NOT-OD-14-073.html, NOT-OD-15-106 at https://grants.nih.gov/grants/guide/notice-files/NOT-OD-15-106.html, and NOT-OD-18-115 at https://grants.nih.gov/grants/guide/notice-files/NOT-OD-18-115.html, including removal of the application from immediate review.

CHAIRPERSON(S)

RICHARDSON, LYNNE D, MD
PROFESSOR AND VICE CHAIR
DEPARTMENT OF EMERGENCY MEDICINE
ICAHN SCHOOL OF MEDICINE AT MOUNT SINAI
NEW YORK, NY 10029

MEMBERS

BIROS, MICHELLE HELEN, MD
ERNEST AND BERNICE RUIZ PROFESSOR
DEPARTMENT OF EMERGENCY MEDICINE
SCHOOL OF MEDICINE
UNIVERSITY OF MINNESOTA
MINNEAPOLIS, MN 55414

BOSSARTE, ROBERT M, PHD *
DIRECTOR, INJURY CONTROL RESEARCH CENTER
DEPARTMENT OF BEHAVIORAL MEDICINE AND PSYCHIATRY
WEST VIRGINIA UNIVERSITY
MORGANTOWN, WV 26506

CABANA, MICHAEL D, MD
PROFESSOR
DEPARTMENT OF PEDIATRICS
ALBERT EINSTEIN COLLEGE OF MEDICINE
BRONX, NY 10467

CAI, XUEYA, PHD *
RESEARCH ASSOCIATE PROFESSOR
DEPARTMENT OF BIOSTATISTICS
AND COMPUTATIONAL BIOLOGY
UNIVERSITY OF ROCHESTER MEDICAL CENTER
ROCHESTER, NY 14642

CHEVILLE, ANDREA LYNNE, MD *
PROFESSOR
DEPARTMENT OF PHYSICAL MEDICINE
AND REHABILITATION
MAYO CLINIC
ROCHESTER, MN 55905

CHOO, ESTHER K, MD
PROFESSOR
DEPARTMENT OF EMERGENCY MEDICINE
OREGON HEALTH AND SCIENCE UNIVERSITY
PORTLAND, OR 97239

GAREEN, ILANA F, PHD
ASSOCIATE PROFESSOR
DEPARTMENT OF EPIDEMIOLOGY
CENTER FOR STATISTICAL SCIENCES
SCHOOL OF PUBLIC HEALTH
BROWN UNIVERSITY
PROVIDENCE, RI 02912

GOBA, GELILA K, MD *
ASSOCIATE PROFESSOR
DEPARTMENT OF OBSTETRICS AND GYNECOLOGY
COLLEGE OF MEDICINE AT CHICAGO
UNIVERSITY OF ILLINOIS
CHICAGO, IL 60612

GOYAL, MONIKA KUMARI, MD
ASSOCIATE PROFESSOR
DEPARTMENT OF EMERGENCY MEDICINE AND TRAUMA
GEORGE WASHINGTON UNIVERSITY
WASHINGTON, DC 20010

HERNANDEZ, INMACULADA, PHARMD, PHD *
ASSOCIATE PROFESSOR
DIVISION OF CLINICAL PHARMACY
SKAGGS SCHOOL OF PHARMACY
AND PHARMACEUTICAL SCIENCES
UNIVERSITY OF CALIFORNIA, SAN DIEGO
LA JOLLA, CA 92093

JOHNSON, HEATHER M, MD
ASSOCIATE PROFESSOR
CARDIOLOGY/PREVENTIVE CARDIOLOGY
CHRISTINE E. LYNN WOMEN'S HEALTH & WELLNESS INST
BAPTIST HEALTH MEDICAL GROUP NORTH
FLORIDA ATLANTIC UNIVERSITY
BOCA RATON, FL 33486

KENDZOR, DARLA ELIZABETH, PHD
DIRECTOR, HEALTH PROMOTION RESEARCH CENTER
DEPARTMENT OF FAMILY AND PREVENTIVE MEDICINE
OKLAHOMA TOBACCO RESEARCH CENTER
UNIVERSITY OF OKLAHOMA HEALTH SCIENCES CENTER
OKLAHOMA CITY, OK 73104

LEE, YOUNG JI, PHD *
ASSISTANT PROFESSOR
DEPARTMENT OF HEALTH AND COMMUNITY SYSTEMS
SCHOOL OF NURSING; DEPARTMENT OF BIOMEDICAL
INFORMATICS; SCHOOL OF MEDICINE
UNIVERSITY OF PITTSBURG
PITTSBURGH, PA 15213

LUO, XIANGHUA, PHD *
PROFESSOR
DIVISION OF BIOSTATISTICS
SCHOOL OF PUBLIC HEALTH
UNIVERSITY OF MINNESOTA
MINNEAPOLIS, MN 55455

MEURER, WILLIAM JOSEPH, MD
ASSOCIATE PROFESSOR
DEPARTMENT OF EMERGENCY MEDICINE AND NEUROLOGY
UNIVERSITY OF MICHIGAN
ANN ARBOR, MI 48109

MILLER, DAVID P, MD
PROFESSOR
DEPARTMENT OF MEDICINE AND PUBLIC HEALTH
SCIENCES
WAKE FOREST SCHOOL OF MEDICINE
WINSTON-SALEM, NC 27157

MYERS, LAURA CHRISTINE, MD *
RESEARCH SCIENTIST
DIVISION OF RESEARCH
KAISER PERMANENTE NORTHERN CALIFORNIA
OAKLAND, CA 94612

PROFIT, JOCHEN, MD
ASSOCIATE PROFESSOR OF PEDIATRICS AND DIRECTOR
PEDIATRICS - NEONATAL AND DEVELOPMENTAL MEDICINE
STANFORD UNIVERSITY
STANFORD, CA 94305

SARPONG, DANIEL F, PHD
SENIOR RESEARCH SCIENTIST
DEPARTMENT OF GENERAL INTERNAL MEDICINE
SCHOOL OF MEDICINE
YALE UNIVERSITY
NEW HAVEN, CT 06510

SCHAUER, DANIEL P, MD *
ASSOCIATE PROFESSOR OF MEDICINE
DEPARTMENT OF INTERNAL MEDICINE
UNIVERSITY OF CINCINNATI
CINCINNATI, OH 45267

SETOGUCHI-IWATA, SOKO, MD, DRPH *
PROFESSOR OF MEDICINE
DEPARTMENT OF MEDICINE
RUTGERS ROBERT WOOD JOHNSON MEDICAL SCHOOL
NEW BRUNSWICK, NJ 08901

SHAFI, TARIQ, MBBS, MHS
PROFESSOR OF MEDICINE
PROFESSOR OF POPULATION HEALTH
PROFESSOR OF PHYSIOLOGY AND BIOPHYSICS
UNIVERSITY OF MISSISSIPPI MEDICAL CENTER
JACKSON, MS 39216

SHAH, MAUNANK, MD, PHD *
ASSOCIATE PROFESSOR
DIVISION OF INFECTIOUS DISEASES
SCHOOL OF MEDICINE
JOHNS HOPKINS UNIVERSITY
BALTIMORE, MD 21287

SHARRIEF, ANJAIL Z, MD *
ASSOCIATE PROFESSOR
DEPARTMENT OF NEUROLOGY
MCGOVERN MEDICAL SCHOOL
THE UNIVERSITY OF TEXAS HEALTH SCIENCE CENTER
HOUSTON, TX 77030

SHERMAN, SCOTT E, MD
PROFESSOR
DEPARTMENT OF POPULATION HEALTH, MEDICINE
AND PSYCHIATRY
NEW YORK UNIVERSITY SCHOOL OF MEDICINE
NEW YORK, NY 10016

SMID, MARCELA, MD *
ASSISTANT PROFESSOR
DEPARTMENT OF OBSTETRICS AND GYNECOLOGY
UNIVERSITY OF UTAH
SALT LAKE CITY, UT 84132

STARAS, STEPHANIE A S, PHD *
ASSOCIATE PROFESSOR
DEPARTMENT OF HEALTH OUTCOMES
AND BIOMEDICAL INFORMATICS
COLLEGE OF MEDICINE
UNIVERSITY OF FLORIDA
GAINESVILLE, FL 32610

TOLEDO, PALOMA, MD, MPH *
ASSISTANT PROFESSOR OF ANESTHESIOLOGY
DEPARTMENT OF ANESTHESIOLOGY
FEINBERG SCHOOL OF MEDICINE
NORTHWESTERN UNIVERSITY
CHICAGO, IL 60611

WATKINS, KATHERINE E, MD *
SENIOR NATURAL SCIENTIST
RAND CORPORATION
SANTA MONICA, CA 90407

YE, FEI, MPH, PHD *
ASSOCIATE PROFESSOR
DEPARTMENT OF BIOSTATISTICS
VANDERBILT UNIVERSITY MEDICAL CENTER
NASHVILLE, TN 37232

ZARZAUR, BEN L, MD
ENDOWED CHAIR AND PROFESSOR
DEPARTMENT OF SURGERY
UNIVERSITY OF WISCONSIN SCHOOL OF MEDICINE
MADISON, WI 53704

ZHANG, JIAJIA, PHD *
PROFESSOR
DEPARTMENT OF EPIDEMIOLOGY AND BIOSTATISTICS
ARNOLD SCHOOL OF PUBLIC HEALTH
UNIVERSITY OF SOUTH CAROLINA
COLUMBIA, SC 29208

MAIL REVIEWER(S)

BEITELSHEES, AMBER L, MPH, PHARMD, PHMD
ASSOCIATE PROFESSOR
ENDOCRINOLOGY, DIABETES AND NUTRITION DIVISION
UNIVERSITY OF MARYLAND SCHOOL OF MEDICINE
BALTIMORE, MD 21201

SCIENTIFIC REVIEW OFFICER

THRASHER, ANGELA DENISE, PHD
SCIENTIFIC REVIEW OFFICER
CENTER FOR SCIENTIFIC REVIEW
NATIONAL INSTITUTES OF HEALTH
BETHESDA, MD 20892

EXTRAMURAL SUPPORT ASSISTANT

JONES, BELINDA
EXTRAMURAL SUPPORT ASSISTANT
CENTER FOR SCIENTIFIC REVIEW
NATIONAL INSTITUTES OF HEALTH
BETHESDA, MD 20892

* Temporary Member. For grant applications, temporary members may participate in the entire meeting or may review only selected applications as needed.

Consultants are required to absent themselves from the room during the review of any application if their presence would constitute or appear to constitute a conflict of interest.
